# Large Granular Lymphocyte Expansion in Myeloid Diseases and Bone Marrow Failure Syndromes: Whoever Seeks Finds

**DOI:** 10.3389/fonc.2021.748610

**Published:** 2021-10-01

**Authors:** Bruno Fattizzo, Valentina Bellani, Raffaella Pasquale, Juri Alessandro Giannotta, Wilma Barcellini

**Affiliations:** ^1^ Department of Oncology and Hemato-Oncology, University of Milan, Milan, Italy; ^2^ Hematology Unit, Fondazione IRCCS Ca’ Granda Ospedale Maggiore Policlinico, Milan, Italy

**Keywords:** large granular lymphocyte, myelodysplastic syndromes, acute myeloid leukemia, myeloproliferative neoplasm, aplastic anemia

## Abstract

Large granular lymphocytes (LGL) are lymphoid cells characterized by either a T-cell or a natural killer phenotype whose expansion may be reactive to toxic, infectious, and neoplastic conditions, or result from clonal selection. Recently, the higher attention to LGL clones led to their detection in many clinical conditions including myeloid neoplasms and bone marrow failures. In these contexts, it is still unclear whether LGL cells actively contribute to anti-stem cell autoimmunity or are only a reaction to dysplastic/leukemic myelopoiesis. Moreover, some evidence exists about a common clonal origin of LGL and myeloid clones, including the detection of STAT3 mutations, typical of LGL, in myeloid precursors from myelodysplastic patients. In this article we reviewed available literature regarding the association of LGL clones with myeloid neoplasms (myelodysplastic syndromes, myeloproliferative neoplasms, and acute myeloid leukemias) and bone marrow failures (aplastic anemia and pure red cell aplasia, PRCA) focusing on evidence of pathogenic, clinical, and prognostic relevance. It emerged that LGL clones may be found in up to one third of patients, particularly those with PRCA, and are associated with a more cytopenic phenotype and good response to immunosuppression. Pathogenically, LGL clones seem to expand after myeloid therapies, whilst immunosuppression leading to LGL depletion may favor leukemic escape and thus requires caution.

## Introduction

Large granular lymphocytes (LGL) are lymphoid cells characterized by either a T-cell or a natural killer (NK) phenotype that physiologically participate in innate immunity and immunosurveillance. Their expansion may be a response to toxic, infectious, and neoplastic conditions, or result from clonal selection (1). The latter may rarely lead to the development of a lymphoproliferative disorder, namely a T-cell or NK lymphoma with variable aggressiveness. Beyond overt lymphoproliferative disease, the increasing awareness about LGL cells and their phenotype led to the discovery of many clinical associations including idiopathic cytopenias and hematologic malignancies (2, 3). The former are part of a spectrum ranging from peripheral autoantibody mediated cytopenias (autoimmune hemolytic anemia, immune thrombocytopenia, and autoimmune neutropenia) to bone marrow failures (aplastic anemia, AA, and low risk myelodysplastic syndromes) characterized by central immune attack towards stem cells (2). In this context it is still unclear whether LGL cells actively contribute to anti-stem cell autoimmunity or are only part of the proinflammatory microenvironment. Regarding hematologic malignancies, LGL clones have been recently detected in myeloid neoplasms such as myeloproliferative neoplasms and acute myeloid leukemia (1, 2). Whether LGL expansion is only a reactive phenomenon or has a common clonal origin with the myeloid clone is object of open investigation. In this review we collect more recent literature about the association of LGL with myeloid neoplasms and bone marrow failures focusing on evidence of pathogenic, clinical, and prognostic relevance.

## Definition and Detection of LGL Clones

Morphologically, LGL are more than twice the diameter of erythrocytes, and are characterized by mature chromatin, excessive cytoplasm, with or sometimes without prominent cytoplasmic granules. Normally, LGLs comprise 10 to 15% of blood mononuclear cells which may be either surface CD3+ (T-cell) or surface CD3– (NK cell). Most normal LGLs in the peripheral blood are NK cells, whilst some are T lymphocytes (2). As mentioned before, LGLs may configure heterogeneous disorders comprising non-clonal reactive processes, indolent clonal proliferative disorders and highly aggressive neoplasms. World Health Organization (WHO) divides clonal LGL expansions into three disorders: T-cell LGL leukemia (T-LGLL), chronic lymphoproliferative disorders of NK cells (CLPD-NK), and aggressive NK-cell leukemia (ANKL) which is associated with Epstein-Barr virus (EBV) infection of the neoplastic NK cells. In contrast to ANKL, both T-LGLL and CLPD-NK are clinically indolent and have a low risk of transformation into an aggressive malignancy (4). In the last decade, European and US Registry are actively studying LGL leukemias and accumulating evidence on clinical features and outcome. Overall, incidence of LGL leukemia is reported as 0.2-0.72 per million persons per year, with no gender effect, and more than 85% of cases are the T-LGL subtype. Median age at diagnosis is 60 years and the disease is only rarely observed in the infancy (5–7).

Flow cytometry is the gold standard for LGLs detection and is usually based on the expression of NK-associated markers CD16 and CD57. CD56 is another marker, constitutively expressed by circulating normal NK cells and usually downregulated in CLPD-NK; its expression in T-LGLL may be associated with a less-favorable prognosis (2, 3). T-LGLs usually express CD3+, TCR αβ+, CD4−, CD5dim, CD8+, CD16+, CD27−, CD28−, CD45R0−, CD45RA+, and CD57+ phenotype, representing a constitutively activated phenotype. Less commonly, T-LGLs mount CD4 with variable expression of CD8. The rare CD3+/CD56+ T-LGL leukemias may show higher clinical aggressiveness. T-LGL usually harbor the T-cell receptor (TCR) αβ+ heterodimer, rarely γδ TCR heterodimer. NK-LGLs are characterized by CD2+/sCD3-/CD3ε+/TCRαβ-/CD4-/CD8+/CD16+/CD56+ phenotype. Evidence of T-LGL clonality is assessed using TCRγ-polymerase chain reaction analyses (PCR) and deep sequencing of TCR has demonstrated a restricted diversity of TCR repertoire. Vβ TCR gene repertoire analysis can also be ascertained using flow cytometry, although this is not routinely performed (8). NK-LGLs do not express TCR so it is difficult to assess their clonality. However, they often show abnormal killer immunoglobulin-like receptor (KIR) expression with complete absence of surface KIR or restricted expression. Restricted KIR expression is often seen in both in T- and NK-LGL leukemia (2).

Regarding other markers, LGL leukemia patients show increased serum levels of interferon-γ 2, monocyte chemoattractant protein-1 (attractive factor for monocytes, T, and NK cells to sites of inflammation), epidermal growth factor, and various interleukins (IL) including IL-6, IL-8, and IL-18. Rheumatoid factor and antinuclear antibody are detected in 60% and 40% of patients, respectively (2). Serum protein electrophoresis usually shows polyclonal hypergammaglobulinemia. Defects in downregulation of Ig secretion in LGL leukemia could explain part of association with autoantibodies malignancies (3).

## Pathogenesis of LGL Expansion

From a pathogenic perspective, it is generally thought that normal LGLs acquire a defect of apoptosis that leads to their accumulation. An interesting explanation for this phenomenon is the expansion of an oligoclonal LGL population under chronic stimulation from an unknown antigen. LGL cells may then acquire a molecular lesion promoting monoclonal proliferation, and release cytokines and toxic granules that contribute to bone marrow failure (2). Concerning apoptosis, LGL cells strongly express Fas (CD95) and Fas-ligand (Fas-L) (CD178). Moreover, RAS and ERK constitutive activation and G12 KRAS mutation are often found in NK-LGL leukemia, and their blockade may restore Fas sensitivity in leukemic LGLs. Although not routinely performed, increased soluble Fas-L is a good surrogate marker of LGL leukemia (9). From a cytogenetic point of view, karyotype is normal in most cases. Recurrent somatic mutations in the Src homology 2 (SH2) domain of the signal transducer and activator of transcription 3 (STAT3) gene have been found in 27-40% of patients with T-LGL leukemia and 30% of patients with CLPD-NK. These mutations lead to constitutive activation of STAT3, with consequent dysregulation of genes downstream of STAT3 (10). Once dimerized, STAT3 shuttles from the cytoplasm to the nucleus, where it ultimately binds to DNA, mediating growth and survival. Disease manifestations such as cytopenias and autoimmune diseases may result from the production of proinflammatory cytokines mediated by STAT3 hyperactivation, as well as from a direct attack on bone marrow by the STAT3-activated LGL (11–13).

Another relevant member of STAT protein family is STAT5b which has been reported to carry gain-of-function mutations in 15–55% of CD4+ T-LGLL, and in 19% of TCRγδ LGLL (14, 15). STAT5b N642H has been identified as an oncogenic driver in innate-like lymphocytes, and a mouse model expressing human N642H mutated STAT5b developed severe CD8+ T-cell neoplasia. IL-15 is an upstream factor of STAT5b and seems crucial for neoplastic transformation. In fact, IL-15 transgenic mice developed the aggressive variant of T or NK cell leukemia (15). The requirement of additional cytokine signals on STAT5b genetic lesions to lead neoplastic evolution suggests the importance of the immunological microenvironment. STAT3 and STAT5b mutations have been included in the 2017 WHO classification of LGLL and STAT5b mutation is associated with a more aggressive clinical course (16). Another gene recurrently mutated in LGLL is TNFα-induced protein 3 (TNFAIP3), a tumor suppressor encoding A20, a negative regulator of nuclear factor kappa B (NFkB) (17). Other genes occasionally mutated in T-LGLL, mainly linked to STAT3 signaling pathway and cytotoxic T lymphocyte activation, are PTPRT, BCL11B, PTPN14, PTPN23 (15). Moreover, it has been shown that patients lacking STAT mutations may harbor other lesions involving genes connecting STAT with Ras/MAPK/ERK and IL-15 signaling, such as FLT3, ANGPT2, KDR/VEGFR2, and CD40LG (18). Finally, whole exome sequencing (WES) on 3 STAT-mutation negative CLPD-NK patients found somatic mutations including KRAS, PTK2, NOTCH2, CDC25B, HRASLS, RAB12, PTPRT, and LRBA (15). Altogether, these data shows that LGL clonal selection and expansion result from a complex interplay among genetic and environmental factors that may be heterogeneously combined.

## LGL Clones Reactive to Autoimmune, Infectious, and Other Conditions

LGL clones can be identified in different conditions such as autoimmune diseases, infections, and transplant, likely representing an unbalanced response to systemic infections and/or immune deregulation. It is difficult to differentiate primary LGL leukemia from reactive LGL expansions. Flow cytometry patterns, together with the molecular lesions, are important tools to assess “quantity and quality” of LGL populations and establish clonality.

### Autoimmune Diseases

Concerning LGL in the context of autoimmune diseases, rheumatoid arthritis is the most common association, being present in up to 18% of patients with LGL expansion (19). This association may be difficult to distinguish from Felty syndrome (FS) that is characterized by chronic arthritis, splenomegaly, and neutropenia, in the setting of longstanding seropositive rheumatoid arthritis. Clonal proliferations of LGLs have been observed in patients with FS, and it has been proposed that these patients may in fact have T-LGLL (20). Clonality tests may be useful, although the patient is generally managed according to the prevailing phenotype (autoimmune versus proliferative). Systemic lupus erythematosus, Sjogren syndrome, autoimmune thyroiditis, autoimmune coagulopathies, vasculitis with cryoglobulinemia, and inclusion body myositis have also been reported as associated with LGLs (2, 19, 21). Overall, autoimmune diseases should be taken into account in the workup of patients with LGL expansion and vice versa.

### Infections

Infections, particularly viral and chronic ones, represent a persistent trigger stimulating lymphocytes with the possible development of lymphoproliferative disorders (22). Cases of LGL expansion secondary to Epstein Bar virus (EBV), cytomegalovirus (CMV), Hepatitis B virus (HBV), Hepatitis C virus (HCV), and Human immunodeficiency virus (HIV) have been reported (2). Moreover, some case reports described untreated strongyloidiasis as cause of chronic inflammation and consequent LGL expansion (23). History of infection and serology for hepatitis and herpetic viruses and HIV have to be investigated when approaching patients with LGL expansion and lymphoproliferative disorders in general.

### Transplant

LGL clones may also arise after both solid and hematopoietic stem cell transplant (HSCT) (24, 25). These procedures induce an immunological storm encompassing the host and the donor immune system. Moreover, the occurrence of viral infections (CMV, EBV, etc.) and the immunosuppressive drugs administered may favor autoimmunity (26). In the case of HSCT, the graft shows immune competence and may mount a response against persistent self-antigens. Graft versus host disease (GVHD) is a typical manifestation, and other autoimmune conditions may develop during immune reconstitution. It has been reported that up to 20% patients show increased LGLs after HSCT, with a median onset of 312 days from transplant, and CMV reactivation and acute GVHD as prominent risk factors (24, 26). Post-transplant LGL expansion, although mainly chronic and indolent, deserves proper investigation in patients with new-onset persistent cytopenia following transplant, since may require adjustment of ongoing immunosuppressive therapy.

### Other Associations

LGL clones are not only associated to autoimmunity but even to immunodeficiency. Although pediatric cases of LGL disorders are rare, a phenotypic overlap may occur with primary immunodeficiencies characterized by increased susceptibility to infections, autoimmunity, and development of lymphoproliferative disorders. Interestingly, LGL clones have been reported in patients with adenosine deaminase 2 deficiency (27). Their presence was related with an activation of phosphatidylinositol-3-phosphate kinase pathway, whose disruption has been implied in the apoptosis imbalance typical of LGL.

Many drugs induce immune/inflammatory perturbations and LGL expansion after the tyrosine kinase inhibitor (TKI) dasatinib (a drug used in chronic myeloid leukemia and Philadelphia positive acute lymphoblastic leukemia) has been reported. Although most LGL clones developing upon dasatinib treatment are asymptomatic, some cases of fever, colitis, and pleural effusions have been reported, suggesting an aberrant immune response (28). Finally, LGLs have been reported after solid tumors and hematologic diseases, particularly myeloid malignancies and bone marrow failure syndromes, as discussed thereafter, and may be associated with autoimmune/autoinflammatory phenomenon such as livedoid vasculopathy, urticarial vasculitis, or complex recurrent aphthous stomatitis in these patients (2).

## LGL Expansion in Myelodysplastic Syndromes

Various evidence exists about LGLs expansion in patients with myelodysplastic syndromes (MDS). Some studies only reported the prevalence of LGLs clones in patients affected by MDS without the development of an overt lymphoproliferative syndrome, whilst other also described a “true” LGL chronic expansion in these subjects (13, 29–33). These findings are summarized in **Table 1**. The prevalence of LGL clones in MDS was highly variable across studies and ranged from 1.4% to 49%. Conversely, in a study by Huh et al., 9 out of 28 patients with T-LGLL also had MDS, and all of them had monoclonal TCR gene rearrangement. Clinically, LGL clones were associated with more marked cytopenias, mainly anemia and thrombocytopenia (30). In particular, patients with T-LGLL/MDS showed lower median hemoglobin and lymphocyte counts when compared with the subgroup affected by T-LGLL alone, whilst platelets levels and neutrophil count were similar (33). Contrarily, in another case series, 9 patients with LGL expansion/MDS from a group of 100 cytopenic subjects showed no significant differences in the grade of cytopenia as compared to patients with MDS or T-LGLL alone (29). These features are consistent with the variable and multifaceted factors contributing to cytopenias in subjects with MDS. In fact, MDS patients are usually elderly, with reduced stem cell reserve, and with pro-inflammatory and pro-apoptotic bone marrow milieu as compared to T-LGLL patients. On the other hand, the presence of a T-LGL infiltrate may contribute to the immune imbalance typical of MDS pathogenesis. In this view, various studies reported the pathogenic role of LGL clones in bone marrow failures and also showed the possibility of a common origin of the two clones. In particular, Durrani et al. analyzed 240 patients with LGL leukemia and found that 5.4% of them was affected by MDS (11/13 with TCR gene rearrangement) (33). They showed that somatic STAT3/STAT5 mutations can be found in up to 15% of LGLL/MDS patients versus 39% of those with LGL clones only. More recently, STAT3-mutated clones were reported in up to 37.5% patients with MDS harboring LGL clones and in 2.5% of MDS alone (13). The detection of LGL-related mutations in MDS cases supports a common pathogenic origin of the two conditions. Interestingly, constitutive STAT5 activation is observed in various myeloid diseases, including chronic myeloid leukemia and JAK2 mutated myeloproliferative syndromes. In fact, JAK/STAT pathway is downstream of many growth factor receptors including those of erythropoietin and thrombopoietin. STAT5b mutations have been associated with more aggressive LGLL phenotype, and recent evidence suggests their unique distribution in T-LGL cells of advanced myeloid neoplasms (35). In another study including 1177 patients with MDS, a LGL clone was found in 322 subjects (27%), and LGL leukemia in 36 (2%). They observed that mutations in certain genes associated with myeloid disorders (e.g., TET2, SF3B1 and ASXL1) had same frequencies in LGL/MDS and MDS alone, whilst U2AF1 mutations were more common among the former (32). Very recently, STAT3 and TET2 mutations were found in 27% and 34% of patients with CLPD-NK, respectively. TET2-mutated CLPD-NK was preferentially associated with MDS, and whole-exome sequencing of sorted cells found that TET2 mutations were shared by myeloid and NK cells indicating that they occurred in early hematopoietic progenitors (36).

**Table 1:** Large granular lymphocytes in myelodysplastic syndromes.

Study Type	Relevance	Main Findings	Ref.
Clinical study	Clinical and therapeutic	11.8% of patients with MDS showed LGL/MDS association and had lower LGL counts and lower response rate to immunosuppression compared to patients with T-LGLL alone.	29
76 MDS, 15 T-LGLL, 9 T-LGLL/MDS.			
Case series	Clinical	Patients with T-LGLL/MDS showed lower median Hb level and lymphocytes compared with patients with T-LGL alone.	30
28 T-LGLL patients, 9 had MDS (32%)			
Clinical study	Pathogenic	Somatic STAT3 mutations may be found in 2.5% of patients affected by MDS, the frequency reaches 37.5% in patients with MDS/LGLL association.	13
367 MDS, 24 with LGL clones (9,2%).			
Clinical study	Clinical and prognostic	85% of LGL/MDS had a TCR gene beta or/and gamma rearrangement by PCR and mainly showed bone marrow hypocellularity. LGL/MDS had similar OS as MDS alone.	31
71 MDS, 35 with MDS/LGL (49%)			
Clinical study 1177 MDS, 322 with LGL clonal expansion (27%)	Pathogenetic/Prognostic	LGL clonal expansion was associated with similar survival and frequency of AML evolution.	32
		TET2, SF3B1 and ASXL1 were the most frequently mutated genes among both groups. U2AF1 mutations more common among LGL/MDS than MDS alone.	
Clinical study	Pathogenic and clinical	5.4% of patients with LGLL had concomitant MDS and were more thrombocytopenic. 15% showed somatic mutations of STAT3/STAT5 versus 39% with LGLL only.	33
240 LGLL, 13/240 (5.4%) had also MDS			
Clinical study	Pathogenic	LGLL/MDS patients were characterized by lower Hb levels and erythroid dysplasia and mostly showed mutations in ASXL1 (30%) and STAG2 (30%).	34
721 MDS, 10 (1.38%) with LGLL			

LGL, large granular lymphocytes; MDS, myelodysplastic syndromes; T-LGLL, T cell large granular lymphocyte leukemia.

From a therapeutic point of view, in the large study by Komrokij et al. MDS patients harboring an LGL clone showed a lower response rate to immunosuppression with anti-thymocyte globulin and cyclosporine as compared to the MDS group (28% *vs* 41%), whilst no difference was observed regarding hypomethylating and erythroid stimulating agents (32). This finding was also confirmed by a different group that showed LGLL/MDS subjects had lower responses to immunosuppression compared to those with T-LGLL alone, possibly due to older age and likely decreased stem cell reserve in those with LGLL/MDS (29). Additionally, it may be speculated that inhibiting two clones may be harder than targeting a single one. Finally, Olson et al. showed that patients with CLPD-NK harboring TET2 mutation show prominent thrombocytopenia and resistance to immunosuppressive treatments (37).

From a prognostic perspective, the presence of an LGL clone did not seem to impact MDS outcome. A study including 71 MDS patients, 49% of whom harbored a T-LGL expansion, did not find substantial differences in OS between the two groups (83 months in the LGL/MDS group versus 65 months in the MDS one) (31). These data were more recently confirmed by Komrokji et al., who found similar median OS (24 months *vs* 27 months) and acute myeloid leukemia (AML) transformation rates (19% in both groups) among patients affected by MDS and LGL/MDS (32).

Concerning overt LGL lymphoproliferative diseases, 3 studies described an association with MDS (30, 33, 34). In particular, Ai et al. evaluated 721 patients with MDS and identified 7 T-LGLL, 2 mixed-phenotype LGLL, and 1 CLPD-NK, resulting in a prevalence of 1.38%. Lower hemoglobin levels, neutropenia and thrombocytopenia were a common finding, as well as a higher frequency of erythroid dysplasia in patients with MDS/LGLL. This condition was mostly associated with mutations in ASXL1 (30%) and STAG2 (30%) genes, and TCR gene rearrangement was present in 9/10 patients (34).

On the whole, LGL expansion may be found in more than 1/3 of MDS patients, is associated with a more cytopenic phenotype, and does not seem to markedly impact on outcome. LGL/MDS cases responded worse to immunosuppression as compared to MDS or LGL alone, so that the same approach as for primary disease is suggested. Conversely, overt LGLL and MDS are rarely associated.

## LGL Expansion in Other Myeloid Neoplasms

LGL clones have been also described in myeloproliferative neoplasms (MPN) and AML, even though this association is rarer (**Table 2**). There are case reports describing the association of LGL clones with AML, acute promyelocytic leukemia (APL), essential thrombocythemia, chronic myeloid leukemia (CML), and chronic myelomonocytic leukemia (38–44). Again, a hallmark of this association is cytopenia. For instance, Reda et al. reported a case of an association between T-LGLL and APL in the same patient, who initially presented with prolonged neutropenia, due to different causes (autoimmunity, APL and LGL expansion). After the APL diagnosis, induction chemotherapy was started, leading to a complete response. Despite chemotherapy, LGLL clone continued to increase, possibly due to a growing advantage after leukemic depletion. Interestingly, severe neutropenia persisted and also interfered with chemotherapy maintenance (44). Finally, even for LGL/AML, it has been speculated that the two clones may origin from the same progenitor (38) and this association did not lead to a worse prognosis. In another experience, Costello et al. reported a 60-year-old man diagnosed with AML, treated with chemotherapy and hematopoietic stem cell transplant with response. Thereafter, the patient developed an LGL expansion requiring therapy with low-dose modified mini-CHOP and methotrexate. Later, AML relapsed, and the patient died (39). Finally, Malani et al. reported a case of concomitant presentation of T-LGLL with AML in an elderly patient who was treated with combination chemotherapy with good outcome (40). Overall, these experiences suggest a relationship among LGL and AML clones: chemotherapy-induced leukemia depletion leads to LGL expansion, whilst immunosuppression reduces immunosurveillance and favors leukemic escape.

**Table 2:** Large granular lymphocytes in acute myeloid leukemia and myeloproliferative neoplasms.

Study Type	Relevance	Main Findings	Ref.
Case report (1 MPN, 1 MDS, 1 HCL)	Pathogenic and prognostic	Clinically, the concomitant existence of LGL proliferation and other leukemia doesn’t seem to be responsible for a worse prognosis on patients.	38
Case report	Pathogenic	LGLs have a significant spontaneous cytotoxicity against autologous leukemia and hematopoietic cells.	39
Case report	Clinical	First reported case of concomitant presentation of T-LGLL with acute myeloid leukemia in an eldery patient who was treated with combination AML chemotherapy and remained alive and well seven months after initial diagnosis.	40
Clinical study	Pathogenic	In the subgroup of patients on dasatinib, clonal lymphocytes increased, both CD8+ cytotoxic cells and NK- and gamma/delta T-cell fractions. These clones may help in the elimination of the residual CML cells.	41
46 CML patients (20 on dasatinib, 14 on imatinib, 12 healthy volunteers			
Case report	Pathogenic	A patient with essential thrombocythemia treated with hydroxyurea. developed a clonal proliferation of cytotoxic T-cells with consequent BM failure	42
Case report	Pathogenic	Concomitant existence of CMML and T-LGL clone may be due to a common pathogenic pathway, linked to immune-dysregulation mediated by expanded cytotoxic T-cells clones.	43
Case report	Clinical	In a patient affected by acute promyelocytic leukemia and concomitant LGL, LGL clones continued to increase despite leukemia chemotherapy. Leukemia treatment may have given a growing advantage to clone expansion of LGL cells.	44

LGL, large granular lymphocytes; AML, acute myeloid leukemia; MPN myeloproliferative syndromes; T-LGLL, T cell large granular lymphocyte leukemia; HCL, hairy cell leukemia; T-LGLL, T cell large granular lymphocyte leukemia; CML, chronic myeloid leukemia; NK, natural killer cells; BM, bone marrow; CMML, chronic myelomonocytic leukemia.

Regarding myeloproliferative diseases, treatment with dasatinib for CML has been associated with an increase in clonal T-/NK-LGL. Kretuzman et al. observed 34 CML patients on treatment with either dasatinib (N=20) or imatinib (N=14): 83% had clonal BCR/ABL-negative lymphocytes (mostly with TCR rearrangement) and this percentage increased during tyrosine kinase therapy with dasatinib but not with imatinib. The Authors speculated that these clones may inhibit the proliferation of residual CML cells and facilitate remission (41). Selvan et al. described a patient with essential thrombocythemia treated with hydroxyurea who eventually developed a clonal proliferation of cytotoxic T-cells and consequent BM failure (42). Finally, concomitant existence of CMML and T-LGLL clone has also been reported with no therapeutic insights (43).

## LGL Expansion in Aplastic Anemia

Many Authors described the association of LGL clones with AA and pure red cell aplasia (PRCA), as summarized in **Table 3**. Some case reports addressed the clinical association only, whilst others speculated about the pathogenic implications. For instance, Handgretinger et al. reported a case of PRCA associated with clonal expansion of T-LGLs of γδ-type. They commented that LGL cells were able to selectively destroy erythroid progenitors that lack HLA class I expression and are unable to inhibit TCR/KIR activation. LGLs contribute to cytopenia by direct toxicity (Fas-FasL interaction) and cytokine production (46). Consistently, Saitoh et al. described polyclonal T-LGLs along with high serum-soluble FasL in a patient who developed severe AA 10 years after Hodgkin lymphoma remission. The patient responded to steroids and cyclosporine, but HL relapsed leading to death. As already discussed for AML, this data indicate that LGL depletion might have impaired immunosurveillance on HL clone (50). More recently, Li et al. analyzed the quantitative and functional changes of CD56bright NK cells in peripheral blood of patients with moderate AA. They found that CD56bright NK were higher than in normal controls, and displayed higher expression of NKG2D and CD158a, likely contributing to disease pathogenesis (53). Zhang et al. compared T lymphocyte subsets in AA and hypoplastic MDS (hypo-MDS) and showed that the proportions of NK- and T-LGL cells in the hypo-MDS group were higher than those in the AA group. These findings indicate that the dysplastic clone (present in hypo-MDS but not in AA) may be a trigger for LGL expansion (52). Finally, PRCA complicated LGL leukemia in 7% of cases only, whilst neutropenia is the leading cytopenia in these patients (48).

**Table 3:** Large granular lymphocytes in aplastic anemia and pure red cell anemia.

Study Type	Relevance	Main Findings	Ref.
Case series	Therapeutic	One patient had PRCA and obtained response with immunosuppressive therapy.	45
10 NK-LGL, 1 PRCA			45
Case series	Therapeutic	PRCA/T-LGLL association predicts superior response to immunosuppressive therapy, but is not correlated with improved survival.	45
47 PRCA, 9 T-LGLL			
Case report	Pathogenic	A case of PRCA with clonal expansion of T-LGLs of γ δ-type in which the malignant LGLs were shown to carry functional inhibitory MHC class I receptors.	46
Case series	Clinical	AA can be a presenting manifestation of T-LGLL, and T-LGLL should be considered in the differential diagnosis of acquired aplastic anemia.	47
9 AA/LGL			
Case series	Pathogenic	14% had pancytopenia at presentation and some fit the diagnostic criteria for AA.	48
203 T-LGLL patients	Therapeutic	LGLL-associated PRCA was observed in 7% of cases and effectively treated with immunosuppression.	
Case report	Therapeutic	T-LGLs of γ δ-type of pure red cell aplasia with low-dose alemtuzumab in a patient with T-LGLs of γ δ-type refractory to cyclosporine and methotrexate	49
Case report	Pathogenic	This case shows a rare instance of a patient who had aplastic anemia associated to polyclonal LGL as the first manifestation of a relapse of Hodgkin lymphoma.	50
Case series	Therapeutic	LGLL with pure red cell aplasia responded well to continuous treatment with cyclophosphamide or cyclosporine A.	51
14 LGLL/PRCA			
Case series	Pathogenic	STAT3 clones can be found in 7% AA and 2.5% MDS and are associated with better responses to immunosuppressive therapy and with HLA-DR15.	13
367 MDS, 140 AA	Clinical		
	Therapeutic		
Case series	Pathogenic	PRCA associated with LGL frequently displays STAT3 mutations.	12
42 T-LGLL, 19 with PRCA (45%), 11			
CLPD-NK, 3 PRCA (27%)			
Case series	Therapeutic	PRCA/LGLL was associated with response to methotrexate. Response was shorter in patients with STAT-3 mutation.	10
36 LGLL, 18 PRCA (50%)			
Case series	Pathogenic	the proportions of NK and T-LGL in the hypoplastic-MDS group were higher than those in the AA group.	52
41 AA, 46 hypoplastic-MDS			
Case series	Pathogenic	STAT3 mutations in 18 of 42 PRCA patients (43%) with or without T-LGLL.	11
54 AA, 21 MDS, 7 PNH, and 42 PRCA		STAT3-mutated CD81 T cells may be closely involved in the selective inhibition of erythroid progenitors in PRCA patients.	
Case series	Pathogenic	CD56bright NK cells in newly diagnosed AA patients was higher than in normal controls	53
50 AA			
Case series	Pathogenic	Somatic mutations in T cells, particularly JAK-STAT and MAPK are frequent in AA patients and may have a pathogenic role.	54
24 AA			

LGL, large granular lymphocytes; AA, aplastic anemia; PRCA, pure red cell anemia; T-LGLL, T cell large granular lymphocyte leukemia; NK, natural killer cells; BM, bone marrow.

At a molecular level, Ishida et al. investigated STAT3 in an Asian cohort of T-LGLL and CLPD-NK of whom a proportion had concomitant PRCA (19/42 and 3/11, respectively). They found STAT3 mutation in 47.6% of T-LGLL and 27.2% of CLPD-NK patients (12). Furthermore, Jerez et al. studied STAT3 mutation in a large series of patients with acquired BMF syndromes, including 140 AA, and identified 16 mutated patients of whom 6 with an LGL clone (13). Other Authors found a STAT3 mutation in 43% of 42 PRCA patients, of whom 13 had associated LGL clones, but not in the 82 patients with AA/MDS (11). More recently, Lundgren et al. showed that CD8+ T cells from AA patients frequently show somatic mutations of JAK-STAT and MAPK pathways, that are associated with CD8+ T-cell clonality and alter CD8+ phenotype (54). On the whole, these studies suggest that STAT3 mutations, may play a pathogenic role, particularly in PRCA, but also in AA, by increasing the production of proinflammatory/proapoptotic cytokines.

Immunosuppression is the backbone therapy for both AA/PRCA and LGL lymphoproliferative disorders, although with heterogeneous outcome (45, 47, 51). In STAT3-mutated AA patients a better response to immunosuppressive therapy has been described in some studies, whilst others reported that STAT3 mutated patients were less responsive to cyclosporine. As regards other immunosuppressants, Lacy et al. reported good response to steroids and azathioprine in an LGLL/PRCA patient (45) and Go et al. described bad responses with cyclophosphamide in LGLL/AA cases (47). More recently, Fujishima et al. analyzed 185 patients with PRCA, of whom 14 had an LGLL clone and responded well to continuous treatment with cyclophosphamide or cyclosporine (51). Finally, methotrexate produced good long-lasting responses in 18 subjects with LGLL/PRCA, similarly to those observed in patients with LGL expansion only (10). Moreover, Schutzinger et al. described successful treatment of LGLL/PRCA with low-dose alemtuzumab, a monoclonal antibody against CD52, in a patient refractory to cyclosporine and methotrexate (49). Finally, only one study addressed the impact of LGL clones on survival of 9 AA/PRCA patients and showed that superior response to immunosuppression did not correlate with improved survival (45). Beyond cytotoxic immunosuppressants, the role of steroids is still controversial, as they may be effective in patients with autoimmune cytopenias associated with LGL-expansion. However, their use in primary LGL, AA, and PRCA is usually not sufficient to revert the phenotype, and association with cytotoxic immunosuppressants is suggested.

In conclusion, this evidence confirms that immuno-suppression, particularly with cyclosporine but also with methotrexate, are good options in bone marrow failures associated with LGL clones. Since AA/PRCA are already treated with cyclosporine combinations, patients with LGL expansion may be effectively treated as the primary disease. A warning persists about infectious risk that represents an important cause of morbidity and mortality in these patients and may be increased by immunosuppression. This may also account for the absence of a favorable effect on survival of LGL/AA association, despite a better response to treatment. Further insights in the pathogenetic mechanisms of LGL/AA associations will possibly enable the development of more targeted and less toxic treatments in the next future (10).

## Discussion and Conclusions

**Figure 1:**
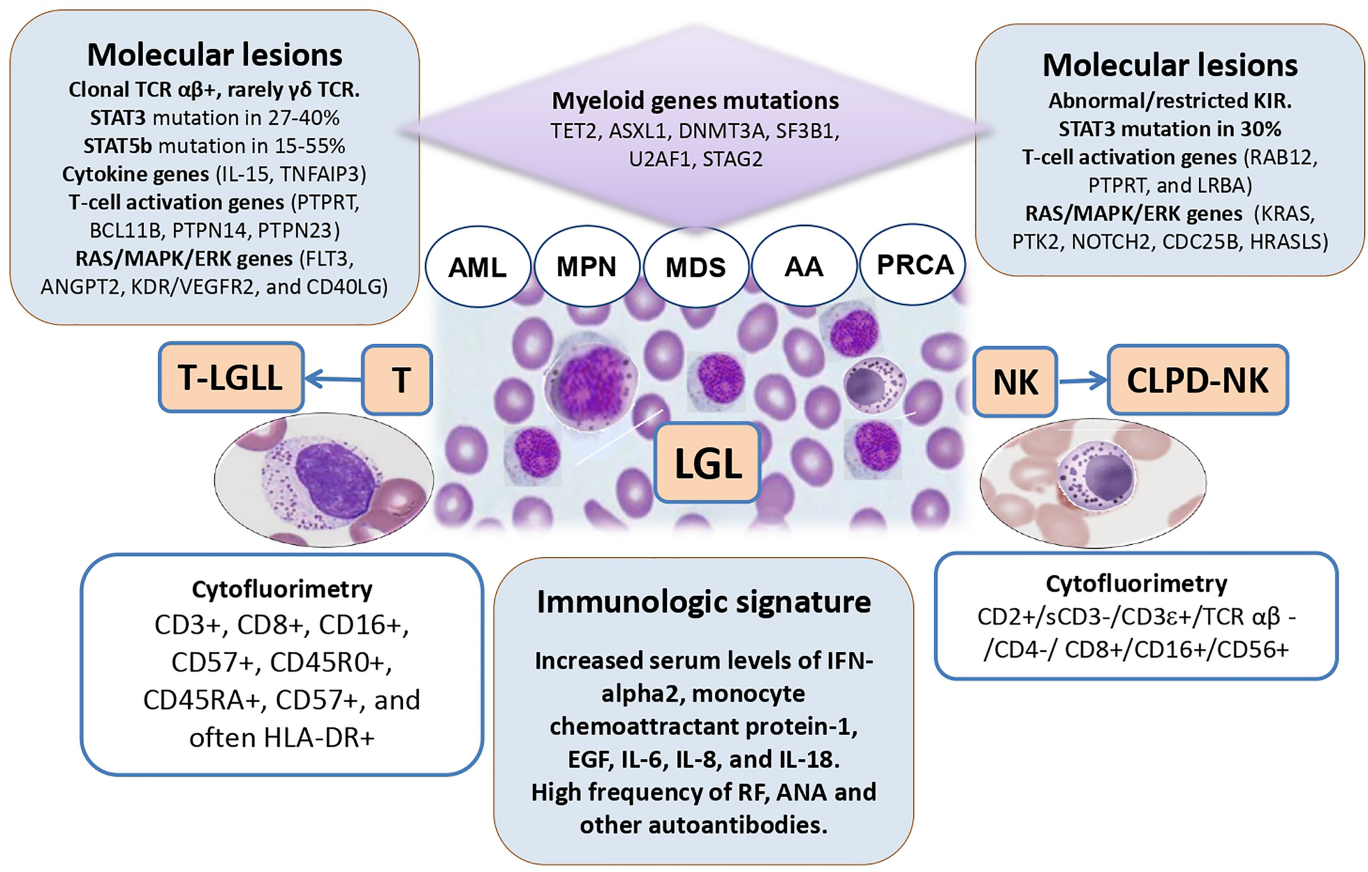
The intersections among large granular lymphocyte (LGL) disorders and myeloid neoplasms. TCR, T-cell receptor; AML, acute myeloid leukemia; MPN, myeloproliferative neoplasm; MDS, myelodysplastic syndromes; AA, aplastic anemia; PRCA, pure red cell aplasia; NK, natural killer cells; T-LGLL, T-cell large granular lymphocyte leukemia; CLPD-NK, chronic lymphoproliferative disorder of NK-cells; IL, interleukin.

LGL clones are increasingly recognized in patients with bone marrow failures (AA/MDS), but also, although rarely, in AML and MPN. **Figure 1** depicts the features and intersections among myeloid neoplasms and LGL disorders. LGL clones are relatively easy to be demonstrated but the communication between the clinician and the pathologist is pivotal for a correct interpretation. In fact, depending on the method utilized (morphology, flow cytometry, TCR/KIR clonality by molecular analysis) the prevalence of LGL clones in these conditions ranges from less than 5% to more than 30%. Their detection is usually associated with a more cytopenic phenotype, likely due to a more pro-inflammatory and pro-apoptotic bone marrow milieu, where LGL clones may induce direct toxicity against stem cells or produce a variety of immunoregulatory cytokines. Moreover, LGL clones are associated with autoimmune phenomena, frequently described in myeloid neoplasms (55). When peripheral lymphocytosis and splenomegaly are present, overt lymphoproliferative disease should be assessed and classified as per WHO criteria. The latter may in fact require specific immunosuppressive treatment. To avoid overtreatment, LGL-specific therapies are indicated based on clinical features of cytopenia (symptomatic anemia, thrombocytopenia, and neutropenia), constitutional symptoms, and lymphoproliferative progression (2). Moreover, it is worth mentioning that splenomegaly, although considered a classic association, has been reported in about 1/5 of patients only, and may be a common finding in several hematologic diseases, including MPN.

Immunosuppression is the mainstay treatment of LGL disorders as well as of AA and hypo-MDS. In these contexts, the presence of an LGL clone seems associated with better outcome, particularly if cyclosporine or methotrexate are used. At variance, patients with MDS/LGL showed a worse treatment outcome as compared with those with LGLL, likely due to an older age and reduced stem cell reserve in the former. An intriguing feature of LGL clones is that they tend to expand after therapy, particularly if they are associated with myeloid disease. This phenomenon is common to other clonal entities such as paroxysmal nocturnal hemoglobinuria (PNH). Here, a multi-step pathogenesis is postulated encompassing the acquisition of a somatic mutation of PIG-A gene, the autoimmune attack to normal stem cells, and the selection/expansion of the PNH clone favored by immunosuppression and/or acquisition of co-mutations (56). Similarly, in LGL clones, somatic mutations of STAT3 and STAT5b have been demonstrated, that seem however not sufficient to cause the disease, without additional contribution of environmental factors, cytokine dysregulation, and therapies. To make this picture even more confusing, the possible common origin of the two clones has been reported, since STAT mutations have been found in myeloid precursors of LGL/MDS patients and myeloid genes mutations have been detected in LGL cells. Overall, although regarded as an important pathogenic player, the detection of a STAT mutation does not inform treatment for LGL that is still based on the severity of cytopenia, B symptoms, and lymphoproliferative progression (2).

The relationship of LGL clones with AA/MDS prognosis is less clear. In fact, some studies indicated a better outcome for LGL/MDS association as compared with MDS alone, whilst other showed no differences despite a better response to immunosuppression. A possible explanation may be the increased infectious risk that represents an important cause of morbidity and mortality after immunosuppression. Furthermore, it may be speculated that LGL depletion after immunosuppression may reduce immunosurveillance on leukemic landscape and counteract the advantage obtained with a better response to immunosuppression. This has been observed even in patients with more aggressive diseases, such as acute myeloid leukemia, where immunosuppression improved LGL signs/symptoms but led to leukemia relapse.

In conclusion, LGL clones may be detected in myeloid diseases in a “whoever seeks finds” fashion. The use of cytofluorimetric and molecular techniques, although the analysis of somatic mutation is not routinely available, will likely allow us to investigate small lymphoid clonalities in myeloid diseases, but even in the general population. This might unveil the presence of a “clonal lymphopoiesis of indeterminate potential” that may precede overt lymphoproliferative diseases, including LGL, but also favor the development of autoimmune diseases still called “idiopathic”.

## Authors Contributions

All authors contributed to the article and approved the submitted version.

## Conflict of Interest

The authors declare that the research was conducted in the absence of any commercial or financial relationships that could be construed as a potential conflict of interest.

## Publisher’s Note

All claims expressed in this article are solely those of the authors and do not necessarily represent those of their affiliated organizations, or those of the publisher, the editors and the reviewers. Any product that may be evaluated in this article, or claim that may be made by its manufacturer, is not guaranteed or endorsed by the publisher.

## References

[1] Oshimi K. Clinical Features, Pathogenesis, and Treatment of Large Granular Lymphocyte. Leukemias (2017) 56(14):1759–69. doi: 10.2169/internalmedicine.56.8881 PMC5548667 28717070

[2] Lamy T Moignet A Loughran T Jr. LGL Leukemia: From Pathogenesis to Treatment. Blood (2017) 129(9):1082–94. doi: 10.1182/blood-2016-08-692590 28115367

[3] Sokol L Loughran TP. Large Granular Lymphocyte Leukemia. Oncologist (2006) 11(3):263–73. doi: 10.1634/theoncologist.11-3-263 16549811

[4] Swerdlow SH Campo E Pileri SA Harris NL Stein H Siebert R. The 2016 Revision of the World Health Organization Classification of Lymphoid Neoplasms. Blood (2016) 127(20):2375–90. doi: 10.1182/blood-2016-01-643569 PMC4874220 26980727

[5] Bareau B Rey J Hamidou M Donadieu J Morcet J Reman O. Analysis of a French Cohort of Patients With Large Granular Lymphocyte Leukemia: A Report on 229 Cases. Haematologica (2010) 95(9):1534–41. doi: 10.3324/haematol.2009.018481 PMC2930955 20378561

[6] Dinmohamed AG Brink M Visser O Jongen-Lavrencic M. Population-Based Analyses Among 184 Patients Diagnosed With Large Granular Lymphocyte Leukemia in the Netherlands Between 2001 and 2013. Leukemia (2016) 30(6):1449–51. doi: 10.1038/leu.2016.68 27055870

[7] Olson KC Moosic KB Jones MK Larkin PMK Olson TL Toro MF. Large Granular Lymphocyte Leukemia Serum and Corresponding Hematological Parameters Reveal Unique Cytokine and Sphingolipid Biomarkers and Associations With STAT3 Mutations. Cancer Med (2020) 9(18):6533–49. doi: 10.1002/cam4.3246 PMC7520360 32710512

[8] Lima M Almeida J Santos AH dos Anjos Teixeira M Alguero MC Queirós ML. Immunophenotypic Analysis of the TCR-Vbeta Repertoire in 98 Persistent Expansions of CD3(+)/TCR-Alphabeta(+) Large Granular Lymphocytes: Utility in Assessing Clonality and Insights Into the Pathogenesis of the Disease. Am J Pathol (2001) 159(5):1861–8. doi: 10.1016/S0002-9440(10)63032-5 PMC1867049 11696446

[9] Liu JH Wei S Lamy T Li Y Epling-Burnette PK Djeu JY. Blockade of Fas-Dependent Apoptosis by Soluble Fas in LGL Leukemia. Blood (2002) 100(4):1449–53. doi: 10.1182/blood.V100.4.1449.h81602001449_1449_1453 12149230

[10] Qiu Z Fan L Wang R Gale RP Liang H Wang L. Methotrexate Therapy of T-Cell Large Granular Lymphocytic Leukemia Impact of STAT3 Mutation. Oncotarget (2016) 7(38):61419–25. doi: 10.18632/oncotarget.11360 PMC5308661 27542218

[11] Kawakami T Sekiguchi N Kobayashi J Imi T Matsuda K Yamane T. Frequent STAT3 Mutations in CD8 1 T Cells From Patients With Pure Red Cell Aplasia. Blood Adv (2018) 2(20):2704–12. doi: 10.1182/bloodadvances.2018022723 PMC6199660 30337298

[12] Ishida F Matsuda K Sekiguchi N Makishima H Taira C Momose K. STAT3 Gene Mutations and Their Association With Pure Red Cell Aplasia in Large Granular Lymphocyte Leukemia. Cancer Sci (2014) 105(3):342–6. doi: 10.1111/cas.12341 PMC4317942 24350896

[13] Jerez A Clemente MJ Makishima H Rajala H Ines G Olson T. STAT3 Mutations Indicate the Presence of Subclinical T-Cell Clones in a Subset of Aplastic Anemia and Myelodysplastic Syndrome Patients. Blood (2013) 122(14):2453–9. doi: 10.1182/blood-2013-04-494930 PMC3790512 23926297

[14] Rajala HL Eldfors S Kuusanmäki H van Adrichem AJ Olson T Lagström S. Discovery of Somatic STAT5b Mutations in Large Granular Lymphocytic Leukemia. Blood (2013) 121(22):4541–50. doi: 10.1182/blood-2012-12-474577 PMC3668487 23596048

[15] Teramo A Barilà G Calabretto G Vicenzetto C Gasparini VR Semenzato G. Insights Into Genetic Landscape of Large Granular Lymphocyte Leukemia. Front Oncol (2020) 10:1–7. doi: 10.3389/fonc.2020.00152 32133291 PMC7040228

[16] Matutes E. The 2017 WHO Update on Mature T- and Natural Killer (NK) Cell Neoplasms. Int J Lab Hematol (2018) 40(Suppl. 1):97–103. doi: 10.1111/ijlh.12817 29741263

[17] Johansson P Bergmann A Rahmann S Wohlers I Scholtysik R Przekopowitz M. Recurrent Alterations of TNFAIP3 (A20) in T-Cell Large Granular Lymphocytic Leukemia. Int J Cancer (2016) 138(1):121–4. doi: 10.1002/ijc.29697 26199174

[18] Coppe A Andersson EI Binatti A Gasparini VR Bortoluzzi S Clemente M. Genomic Landscape Characterization of Large Granular Lymphocyte Leukemia With a Systems Genetics Approach. Leukemia (2017) 31(5):1243–46. doi: 10.1038/leu.2017.49 PMC5419584 28167832

[19] Zhang R Shah MV Loughran TP Jr. The Root of Many Evils: Indolent Large Granular Lymphocyte Leukaemia and Associated Disorders. Hematol Oncol (2010) 28(3):105–17. doi: 10.1002/hon.917 PMC4377226 19645074

[20] Liu X Loughran TP Jr. The Spectrum of Large Granular Lymphocyte Leukemia and Felty’s Syndrome. Curr Opin Hematol (2011) 18(4):254–9. doi: 10.1097/MOH.0b013e32834760fb PMC4377227 21546829

[21] Sun H Wei S Yang L. Dysfunction of Immune System in the Development of Large Granular Lymphocyte Leukemia. Hematology (2019) 24(1):139–47. doi: 10.1080/10245332.2018.1535294 30334691

[22] Yang J Epling-Burnette PK Painter JS Zou J Bai F Wei S. Antigen Activation and Impaired Fas-Induced Death-Inducing Signaling Complex Formation in T-Large-Granular Lymphocyte Leukemia. Blood (2008) 111(3):1610–6. doi: 10.1182/blood-2007-06-093823 PMC2214759 17993614

[23] Rishi MA Chaudhry SZ. Pulmonary Strongyloidiasis Associated CD3+ Large Granular Lymphocytosis. Ann Thorac Med (2011) 6(2):96–8. doi: 10.4103/1817-1737.78432 PMC3081564 21572700

[24] Kim DDH Chang H Panzarella T Gupta V Kuruvilla J Lipton JH. Large Granular Lymphocytosis and Its Impact on Long-Term Clinical Outcomes Following Allo-SCT. Bone Marrow Transplant (2013) 48(8):1104–11. doi: 10.1038/bmt.2013.5 23396405

[25] Alfano G Fontana F Colaci E Mori G Cerami C Messerotti A. T-Cell Large Granular Lymphocyte Leukemia in Solid Organ Transplant Recipients: Case Series and Review of the Literature. Int J Hematol (2019) 110(3):313–21. doi: 10.1007/s12185-019-02682-2 31250283

[26] Nann-r S Tzankov A Cantoni N Heim D Tsakiris D Arber C. Large Granular Lymphocyte Expansion After Allogenic Hematopoietic Stem Cell Transplant Is Associated With a Cytomegalovirus Reactivation and Shows an Indolent Outcome. Biol Blood Marrow Transplant (2012) 18(11):1765–70. doi: 10.1016/j.bbmt.2012.07.007 22796340

[27] Saettini F Fazio G Corti P Quadri M Bugarin C Gaipa G. Two Siblings Presenting With Novel ADA2 Variants, Lymphoproliferation, Persistence of Large Granular Lymphocytes, and T-Cell Perturbations. Clin Immunol (2020) 218:108525. doi: 10.1016/j.clim.2020.108525 32659374

[28] Qiu Z Xu W Li J. Large Granular Lymphocytosis During Dasatinib Therapy. Cancer Biol Ther (2014) 15(3):247–55. doi: 10.4161/cbt.27310 PMC3974824 24352048

[29] Saunthararajah Y Molldrem JJ Rivera M Williams A Stetler-stevenson M Sorbara L. Coincident Myelodysplastic Syndrome and T-Cell Large Granular Lymphocytic Disease: Clinical and Pathophysiological Features. Br J Haematol (2001) 112(1):195–200. doi: 10.1046/j.1365-2141.2001.02561.x 11167802

[30] Huh YO Medeiros LJ Ravandi F Konoplev S Jorgensen JL Miranda RN. T-Cell Large Granular Lymphocyte Leukemia Associated With Myelodysplastic Syndrome A Clinicopathologic Study of Nine Cases. Am J Clin Pathol (2009) 131(3):347–56. doi: 10.1309/AJCP6YHI1JEXAWAP 19228641

[31] Zhang X Sokol L Bennett JM Moscinski LC List A. T-Cell Large Granular Lymphocyte Proliferation in Myelodysplastic Syndromes: Clinicopathological Features and Prognostic Significance. Leuk Res (2016) 43:18–23. doi: 10.1016/j.leukres.2016.02.006 26927701

[32] Komrokji RS Ali NA Sallman D Padron E Lancet J Sokol L. Characterization of Myelodysplastic Syndromes (MDS) With T-Cell Large Granular Lymphocyte Proliferations (LGL). Leukemia (2020) 34(11):3097–9. doi: 10.1038/s41375-020-0928-4 32565544

[33] Durrani J Awada H Kishtagari A Visconte V Kerr C Adema V. Large Granular Lymphocytic Leukemia Coexists With Myeloid Clones and Myelodysplastic Syndrome. Leukemia (2020) 34(3):957–62. doi: 10.1038/s41375-019-0601-y PMC8370475 31624375

[34] Ai K Li M Wu P Deng C Huang X Ling W. Concurrence of Myelodysplastic Syndromes and Large Granular Lymphocyte Leukemia: Clinicopathological Features, Mutational Profile and Gene Ontology Analysis in a Single Center. Am J Cancer Res (2021) 11(4):1616–31. PMC8085857 33948377

[35] Qu S Jia Y Wang H Ai X Xu Z Qin T. STAT3 and STAT5B Mutations Have Unique Distribution in T-Cell Large Granular Lymphocyte Proliferations and Advanced Myeloid Neoplasms. Leuk Lymph (2021) 62(6):1506–09. doi: 10.1080/10428194.2020.1869964 PMC9204507 33410350

[36] Pastoret C Desmots F Drillet G Le Gallou S Boulland ML Thannberger A. Linking the KIR Phenotype With STAT3 and TET2 Mutations to Identify Chronic Lymphoproliferative Disorders of NK Cells. Blood (2021) 137(23):3237–50. doi: 10.1182/blood.2020006721 PMC8351897 33512451

[37] Olson TL Cheon H Xing JC Olson KC Paila U Hamele CE. Frequent Somatic TET2 Mutations in Chronic NK-LGL Leukemia With Distinct Patterns of Cytopenias. Blood (2021) 138(8):662–73. doi: 10.1182/blood.2020005831 PMC8394905 33786584

[38] Bassan R Rambaldi A Allavena P Abbate M Marini B Barbui T. Association of Large Granular Lymphocyte/Natural Killer Cell Proliferative Disease and Second Hematologic Malignancy. Am J Hematol (1988) 29(2):85–93. doi: 10.1002/ajh.2830290206 3263796

[39] Costello RT Sivori S Mallet F Sainty D Arnoulet C Reviron D. A Novel Mechanism of Antitumor Response Involving the Expansion of CD3 +/CD56 + Large Granular Lymphocytes Triggered by a Tumor-Expressed Activating Ligand. Leukemia (2002) 16(5):855–60. doi: 10.1038/sj.leu.2402488 11986947

[40] Malani AK Gupta C Rangineni R Singh J Ammar H. Concomitant Presentation of Acute Myeloid Leukemia With T-Cell Large Granular Lymphocytic Leukemia. Acta Oncol (2009) 46(2):247–9. doi: 10.1080/02841860600827139 17453377

[41] Kreutzman A Juvonen V Kairisto V Ekblom M Stenke L Seggewiss R. Mono/oligoclonal T and NK Cells Are Common in Chronic Myeloid Leukemia Patients at Diagnosis and Expand During Dasatinib Therapy. Blood (2010) 116(5):772–82. doi: 10.1182/blood-2009-12-256800 20413659

[42] Selvan SR Sheehy PF Heinemann FS Anbuganapathi S. Bone Marrow Failure Due to T-Cell Large Granular Lymphocytic Leukemia in a Patient With Essential Thrombocythemia. Leuk Res (2011) 35(2):278–82. doi: 10.1016/j.leukres.2010.09.004 20934219

[43] Song S. Brief Communication A Case Report: Concurrent Chronic Myelomonocytic Leukemia and T-Cell Large Granular Lymphocytic Leukemia-Type Clonal Proliferation as Detected by Multiparametric Flow Cytometry. Citomet B Clin Cytom (2011) 80(2):126–9. doi: 10.1002/cyto.b.20565 21337493

[44] Reda G Fattizzo B Cassin R Flospergher E Orofino N Gianelli U. Multifactorial Neutropenia in a Patient With Acute Promyelocytic Leukemia and Associated Large Granular Lymphocyte Expansion: A Case Report. Oncol Lett (2017) 13(3):1307–10. doi: 10.3892/ol.2016.5549 PMC5403302 28454252

[45] Lacy BMQ Kurtin PJ Tefferi A. Pure Red Cell Aplasia: Association With Large Granular Lymphocyte Leukemia and the Prognostic Value of Cytogenetic Abnormalities. Blood (1996) 87(7):3000–6. doi: 10.1182/blood.V87.7.3000.bloodjournal8773000 8639922

[46] Fisch P Handgretinger R Schaefer HE. Pure Red Cell Aplasia. Br J Haematol (2000) 111(4):1010–22. doi: 10.1046/j.1365-2141.2000.02429.x 11167735

[47] Go RS Tefferi A Li C Lust JA Phyliky RL. Brief Report Lymphoproliferative Disease of Granular T Lymphocytes Presenting as Aplastic Anemia. Blood (2000) 96(10):3644–6. doi: 10.1182/blood.V96.10.3644 11071666

[48] Go R Lust JA Phyliky RL. Aplastic Anemia and Pure Red Cell Aplasia Associated With Large Granular Lymphocyte Leukemia. Semin Hematol (2003) 40(3):196–200. doi: 10.1016/S0037-1963(03)00140-9 12876668

[49] Schützinger C Gaiger A Thalhammer R Vesely M Fritsche-Polanz R Schwarzinger I. Remission of Pure Red Cell Aplasia in T-Cell Receptor Cd -Large Granular Lymphocyte Leukemia After Therapy With Low-Dose Alemtuzumab. Leukemia (2005) 19(11):2005–8. doi: 10.1038/sj.leu.2403956 16193089

[50] Saitoh T Karasawa M Sakuraya M Norio N Junko T Shirakawa K. Improvement of Extrathymic T Cell Type of Large Granular Lymphocyte (LGL) Leukemia by Cyclosporin A: The Serum Level of Fas Ligand is a Marker of LGL Leukemia Activity. Eur J Haematol (2000) 65(4):272–5. (May 1997). doi: 10.1034/j.1600-0609.2000.065004272.x 11073168

[51] Fujishima N Sawada K Hirokawa M Oshimi K Sugimoto K Matsuda A. Long-Term Responses and Outcomes Following Immunosuppressive Therapy in Large Granular Lymphocyte Leukemia-Associated Pure Red Cell Aplasia: A Nationwide Cohort Study in Japan for the PRCA Collaborative Study Group. Haematologica (2008) 93(10):1555–9. doi: 10.3324/haematol.12871 18641028

[52] Zhang H Huang Z Wu X Li Q Yu Z. Comparison of T Lymphocyte Subsets in Aplastic Anemia and Hypoplastic. Life Sci (2017) 189:71–5. doi: 10.1016/j.lfs.2017.09.020 28935248

[53] Li Y Ding S Liu C Chen T Liu H Li L. Abnormalities of Quantities and Functions of CD56bright Natural Killer Cells in Non-Severe Aplastic Anemia. Hematology (2019) 24(1):405–12. doi: 10.1080/16078454.2019.1590963 30907293

[54] Lundgren S Keränen MAI Kankainen M Huuhtanen J Walldin G Kerr CM. Somatic Mutations in Lymphocytes in Patients With Immune-Mediated Aplastic Anemia. Leukemia (2021) 35(5):1365–79. doi: 10.1038/s41375-021-01231-3 PMC8102188 33785863

[55] Barcellini W Giannotta JA Fattizzo B. Autoimmune Complications in Hematologic Neoplasms. Cancers (2021) 13(7):1532. doi: 10.3390/cancers13071532 33810369 PMC8037071

[56] Fattizzo B Ireland R Dunlop A Yallop D Kassam S Large J. Clinical and Prognostic Significance of Small Paroxysmal Nocturnal Hemoglobinuria Clones in Myelodysplastic Syndrome and Aplastic Anemia. Leukemia (2021). doi: 10.1038/s41375-021-01190-9 PMC8550969 33664463

